# Faster Repetition Rate Sharpens the Cortical Representation of Echo Streams in Echolocating Bats

**DOI:** 10.1523/ENEURO.0410-21.2021

**Published:** 2022-02-08

**Authors:** Silvio Macias, Kushal Bakshi, Michael Smotherman

**Affiliations:** 1 Department of Biology, Texas A&M University, College Station, TX 77843; 2 Institute for Neuroscience, Texas A&M University, College Station, TX 77843

## Abstract

There is consensus that primary auditory cortex (A1) utilizes a combination of rate codes and temporally precise population codes to represent discreet auditory objects. During the response to auditory streams, forward suppression constrains cortical rate coding strategies, but it may also be well positioned to enhance temporal coding strategies that rely on synchronized firing across neural ensembles. Here, we exploited the rapid temporal dynamics of bat echolocation to investigate how forward suppression modulates the cortical ensemble representation of complex acoustic signals embedded in echo streams. We recorded from auditory cortex of anesthetized free-tailed bats while stimulating the auditory system with naturalistic biosonar pulse-echo sequences covering a range of pulse emission rates. As expected, increasing pulse repetition rate significantly reduced the number of spikes per echo stimulus, but it also increased spike timing precision and doubled the information gain. This increased spike-timing precision translated into more robust inter-neuronal synchronization patterns with >10-dB higher signal-to-noise ratios (SNRs) at the ensemble level. We propose that forward suppression dynamically mediates a trade-off between the sensitive detection of isolated sounds versus precise spatiotemporal encoding of ongoing sound sequences in auditory cortex.

## Significance Statement

Auditory cortical neurons are unable to follow trains of sounds with repetition rates higher than ∼15 Hz. The dynamics and synaptic mechanisms responsible for this forward suppression are well known. However, their functional consequences on the representation of sounds remain unknown. We evaluated the effects of forward suppression on both rate and temporal coding of complex sounds in auditory cortex. We show that forward suppression can greatly facilitate synchronization-based time codes and thereby enhance the cortical spatiotemporal representation of natural sounds at ecologically relevant rates. Such increases in neuronal synchrony are consistent with emerging theories of how sound spectral envelopes are encoded, with important implications for music and speech processing.

## Introduction

The responses of auditory cortical neurons to preferred sounds decrease over time with repeated stimulation at high rates. This forward suppression of neural activity is evident when one sound diminishes responses to subsequent similar sounds for tens to hundreds of milliseconds (Creutzfeldt et al., 1980; Eggermont, 1992; Brosch and Schreiner, 1997). This widespread phenomenon shapes the neural representation of ongoing complex sound sequences in such ways as preventing individual neurons from faithfully following sound trains faster than ∼15 Hz (Eggermont, 1992). The temporal dynamics of forward suppression are now well described in many species, and recent studies have identified its cellular and synaptic origins (Wehr and Zador, 2005; Bayazitov et al., 2013). However, the theoretical consequences of forward suppression on the cortical representation of sounds remain speculative.

Complex sounds are composed of acoustic features that are independently encoded by the ascending auditory system and subsequently integrated by cortical networks that reconstruct a percept of the complete sound. This is accomplished through a combination of rate coding and time coding strategies in auditory cortex (Nelken et al., 2005). Rate coding captures feature dynamics by the number of evoked spikes per stimulus, while time codes track feature parameters based on relative spike timing across neural ensembles. Forward suppression sharply compresses the dynamic range of spike rates and thereby constrains rate coding (Yin et al., 2011; Malone et al., 2015; Hörpel and Firzlaff, 2019), but its impacts on spike timing or temporal coding schemes are less clear (Schnupp et al., 2006; Yin et al., 2011; Ince et al., 2013). Although there is extensive literature about the use of spike rate to encode temporal parameters, like duration or delay tuning, the bat auditory cortex also uses time coding strategies to cope with the rapid and temporally precise nature of echolocation (Suga, 1989). We took advantage of this to measure the effects of forward suppression on the cortical representation of complex sounds within the context of a computational model of information coding in auditory cortex (Ming et al., 2021).

The bat auditory system is adapted to analyze biosonar echoes (Suga, 1989), which are typically 2- to 3-ms broadband, downward frequency-modulated sweeps. When an echolocation pulse is reflected off of an irregularly shaped surface, multiple overlapping echoes convolve into a single sound endowed with a complex interference pattern of spectral peaks and notches (Simmons et al., 1974). Echo spectral notch patterns are therefore unique to each target, and bats can reconstruct an internal representation of a target’s shape based on these fine spectral details (Schmidt, 1988, 1992; Simmons and Chen, 1989; Ming et al., 2021). The bat auditory cortex contains neurons that preferentially respond with greater spike rates to the presence of unique spectral interference patterns (Sanderson and Simmons, 2002; Firzlaff and Schuller, 2007). However, we recently reported evidence that the cortical representation of target shape may benefit more from the amount of information carried by the spike times, specifically the first spike latency (FSL) of the response of the individual neurons of the bat primary auditory cortex (A1). Theoretically, the observed changes in the response latency of the neurons tuned to the frequency of the notches, usually in the range of 5–8 ms, disrupt or alter the sequential activation along the tonotopic axis of the A1, which leads to an increase of the spike synchrony between neurons tuned to different characteristic frequencies (CFs; Macias et al., 2020). This result in a cortical spatiotemporal spike synchronization pattern that should be unique for each target. Independently derived computational models also predict that bat auditory cortex relies on a precise spike time-dependent synchronization network to reconstruct echoes and classify their source (Saillant et al., 1993; Ming et al., 2021). There is evidence that forward suppression contribute to a sharper rate coding of echo delay (Beetz et al., 2016). Here, we evaluated how forward suppression affects the rate and time coding of target shape in the A1of the echolocating bat. We examined how the reduced firing of the cortical neurons because of the suppression affects the spike synchronization patterns obtained for different targets. In addition, we assessed how forward suppression contributes to the amount of information carried by the changes in the response latency. The results show that forward suppression has the potential to greatly facilitate synchronization-based time codes and thereby enhance the neural representation of natural sounds at ecologically relevant rates.

## Materials and Methods

### Animals

We performed electrophysiological recordings in the A1 of four adult (one female, three males) Mexican free-tailed bats, *Tadarida brasiliensis*. Bats were group housed indoors in an artificial habitat with a reversed light cycle. All animal experimental procedures were conducted in accordance with the National Institutes of Health *Guide for the Care and Use of Laboratory Animals* and were approved by the Institutional Animal Care and Use Committee (IACUC Animal Use Protocol #2017-0163D).

### Surgical procedures

Animals were anesthetized with a solution of sodium pentobarbital (80 mg/kg) and positioned within a custom-built stereotaxic apparatus. Status of anesthesia was monitored by monitoring breathing and ear twitch reflexes and maintained at a surgical plane with supplementary doses as needed. Body temperature was maintained within normal ranges using a heating lamp. The skin and temporal muscles overlying the skull were cut and removed and a custom-fabricated post was attached to the bone at the midline using cyanoacrylate gel. A craniotomy (∼2 × 2 mm) was made using a scalpel blade to expose the left auditory cortex.

### Acoustic stimuli

Acoustic stimuli were digitally synthesized and controlled using a custom-written program in MATLAB (R2018a, MathWorks). Sounds were generated at a sampling rate of 250 kHz with a National Instruments card (NI USB-6356, National Instruments Co). The audio signal was transferred to an audio amplifier (SONY, STR-DE197) and broadcast to the bat with a calibrated ribbon tweeter loudspeaker (Dayton Audio, PTMini-6) centered 10 cm directly in front of the head. The calibration curve was obtained with a Brüel and Kjaer sound recording system (1/4-inch Microphone 4135, Microphone Preamplifier 2670, Brüel and Kjaer) connected to a conditioning microphone amplifier (Nexus 2690, Brüel and Kjaer).

To measure the frequency response area, we presented the animal with a pseudorandomized series of pure tones (10-ms duration, 0.5-ms rise/fall time) at different sound pressure levels (step size 10 dB, range 20–80 dB SPL) and frequencies (step size 5 kHz, range 10–80 kHz). Each frequency-level combination was presented 5 times at an interval of 300 ms. We tested the effect of the temporal arrangement on the cortical representation of the object surfaces. To do this, we ensonified a flat surface and two sandpapers of different grit sizes (60 and 150) with a 3-ms downward FM sweeping between 20 and 70 kHz through an ultrasound speaker (Dayton Audio, PTMini-6) and recorded the returning echoes with a microphone (Brüel and Kjaer, 1/4-inch Microphone 4135, Microphone Preamplifier 2670) located above the speaker. Using recorded the pulses-echoes, we built with sequences of different repetition rates (10, 12, and 15 Hz) for each surface.

### Electrophysiological recordings

Experiments were performed in a custom-built sound-attenuating anechoic chamber. Anesthetized bats were placed in a body mold made of soft plastic foam and the head was tightly affixed to the stereotaxic apparatus by a rod attached to a metal holder. Neuronal recordings were performed using silicon probes from Cambridge Neurotech (16 contacts × 2 shanks per probe with 250 μm between shanks and 50-μm spacing between contact sites along each shank). Each shank had a thickness of 15 μm. Using a micromanipulator system (MX7600R, Siskiyou Corp.), probes were positioned perpendicular to the pial surface based on landmarks and stereotaxic coordinates, and then inserted slowly into the brain through the intact dura mater to a depth of ∼900 ± 50 μm at the deepest contact point. Neuronal data were acquired with an OmniPlex D. Neural Data Acquisition System recording system (Plexon Inc.) at a sampling rate of 40 kHz (per channel) and 16-bit precision. Synchronization between the neural recordings and acoustic stimulus broadcasts was achieved with a TTL pulse output from the National Instrument card and recorded on one of the analog channels of the Plexon data acquisition system.

### Analysis of neural recordings

Since we did not find differences in the frequency tuning, bandwidth of frequency response areas or directional selectivity to the FM sweep across cortical depth (Macias et al., 2019), in this study, we only included data recorded at depths between 400 and 600 μm, corresponding to input Layer IV. The raw signal was digitally bandpass-filtered offline (elliptic, second order) between 500 and 3000 Hz to obtain the multiunit activity. Neural recordings were sorted following methods outlined previously (Quiroga et al., 2004). The Wavelet transformation and the superparamagnetic clustering resulted in isolation of single-unit extracellular potentials that matched with qualitative assessments of spike waveforms and estimates of single-unit isolation based on spike refractory periods. Recordings with spike amplitudes lower than four times the amplitude of the recording background noise were not included in the data analysis. From the raster plots, representing the spike-time versus the trial number, we measured the number of spikes in a window of 50 ms after the stimulus onset for each frequency-level combination to build the frequency response areas. In each frequency response area, we calculated the CF (frequency eliciting the higher number of spikes at the lowest level). In the responses to the CF at 80 dB SPL, we measured the number of spikes and the mean FSL. Because some neurons showed some spontaneous firing, we calculated the mean FSL by measuring the time of the first spike after the poststimulus time histogram reached 25% of its peak. This minimized the influence of spontaneous activity spikes on the response times.

Blood vessels around the medial cerebral artery were very consistent from bat to bat. This allowed us to locate the A1 and use the vessels as reference points for stereotaxic measurements. For each bat, coordinates of the recording sites in relation to a branch of the median cerebral artery were measured using a calibrated micromanipulator (MX7600R, Siskiyou Corp.). All cortices were aligned together for the construction of composite maps using the branches and the median cerebral artery to determine the orientation of the ordinate axis of the bidimensional Cartesian space of analysis.

We calculated synchronization matrices using the response from the population of neurons (Macias et al., 2020) to the echoes from the flat and the two sandpapers surfaces. To build the synchronization matrices, we evaluated spike train synchrony using the spike-synchronization index (*c*), which quantifies the degree of synchrony from the relative number of quasi-simultaneous appearance of spikes. We used SPIKY (Kreuz et al., 2015; Satuvuori et al., 2017), a MATLAB (MathWorks) written graphical user interface for monitoring synchrony between artificially simulated or experimentally recorded neuronal spike trains. Synchronization matrices were calculated using the spike trains elicited in response to the echoes from the different surfaces in all 165 neurons. A matrix was calculated for each trial and from that, we calculated the mean synchronization matrix for each echo.

We evaluate the effect of the forward suppression in the cortical synchronization patterns by calculating the signal-to-noise ratio (SNR) of the synchronization matrices, we considered each matrix as an image and used the matrix calculated in the response to the first pulse-echo pair as the reference. The SNR of each matrix can be expressed as follow:

$$SNR=10\;\log_{10}\left(\frac{x^{2}}{R}\right),$$

where x is the matrix to evaluate and R is the reference matrix (Gonzalez and Woods, 2008).

We calculated mutual information (MI), that quantifies how well an ideal observer of neuronal responses can discriminate between the different stimuli, based on a single response trial (Panzeri et al., 2001; García-Rosales et al., 2018). The MI between a stimulus S and response R can be expressed as follows:

I(R;S)=H(R)−H(R|S),

where H(R) is the response entropy (i.e., the total variability of the response distribution) and is calculated as

$$H\left(R\right)=-\underset{r\in R}{\sum }P\left(r\right)\log\left[P\left(r\right)\right],$$

while

$$H\left(R|S\right)=-\underset{s\in S}{\sum }P(s)\underset{r\in R}{\sum }P\left(r|s\right)\log_{2}[P(r|s)]$$

is known as the “noise entropy” and represents the irreproducibility of the response given a stimulus. The probabilities P(r) and P(s) represent the probability of a particular response in R, and the probability of a particular stimulus in S, respectively, while P(r|s) represents the conditional probability of a response r given a stimulus s. To calculate the neuronal responses (spike rate and mean FSL), we considered a time window of 0–60 ms after the stimulus onset. In calculating the information conveyed only by the spike rate, the response r was computed as the number of spikes emitted in this time window on one trial. To study information conveyed by the FSL, we divided the spike trains into bins of 2 ms. Both P(r) and P(s) depend on the assumptions made regarding how the response is quantified, and how the stimulus set is defined. Note that the units of MI are bits, given that the logarithm used for the calculations is of base 2. Each bit of information implies that an observer can reduce its uncertainty about the stimulus (based on the response) by a factor of 2. All information analyses were conducted using the Information Breakdown Toolbox (ibTB; Panzeri et al., 2001; Magri et al., 2009).

## Results

### Creating naturalistic echo mimic stimuli to measure information coding in auditory cortex

**Figure 1.**
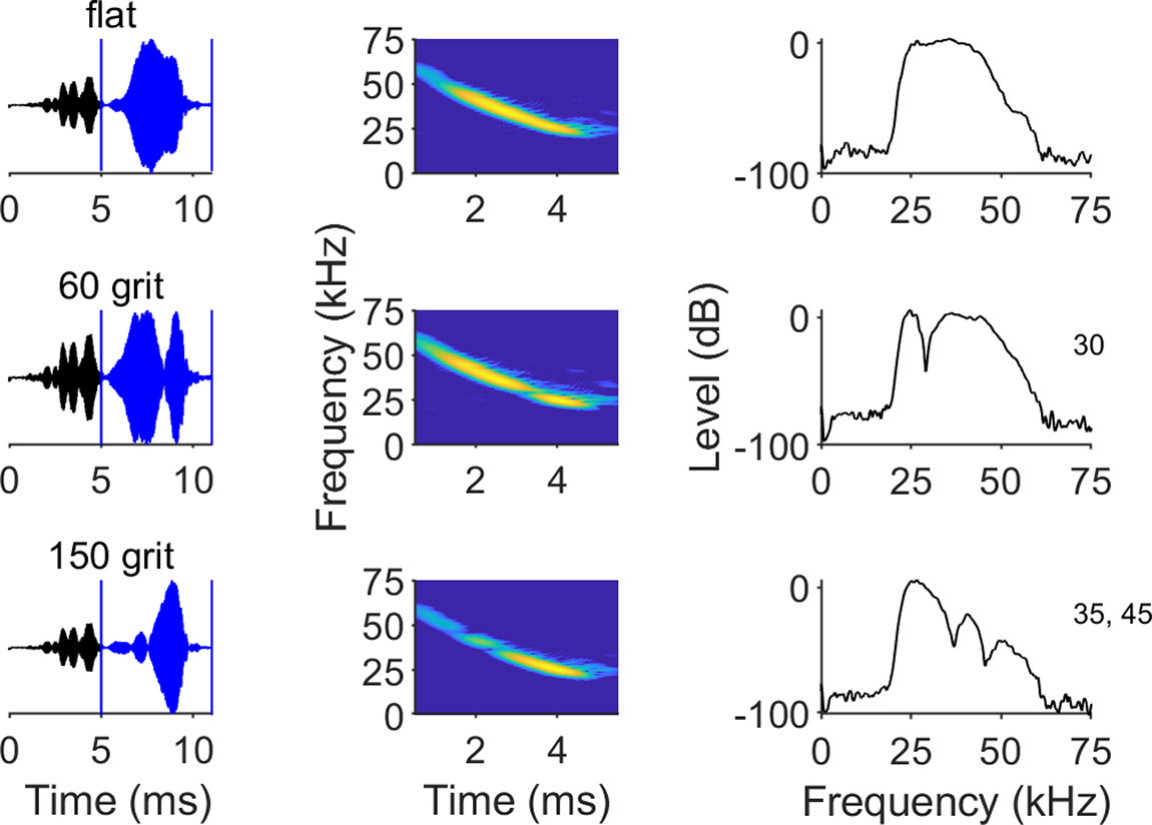
Acoustic stimuli. Oscillogram of the pulse-echo (echo highlighted in blue) pairs and spectrogram and power spectra of the echoes used as stimulus. Insets indicate the frequencies of the amplitude notches in the echoes of surface. The emitted signal was a 3-ms downward frequency-modulated sweep starting at 70 kHz and ending at 20 kHz.

To investigate the effects of forward suppression on the temporal coding of echo spectral envelope, we first needed to create a set of echo mimic acoustic stimuli that we knew the bats could distinguish. Surface texture is an integral cue used by echolocating bats and dolphins for classifying an ensonified target, and previous work showed that bats rely on spectral notch patterns embedded in echoes to resolve textures (Habersetzer and Vogler, 1983; Schmidt, 1988; Simmons et al., 1990). Abrasive sandpapers are available as standardized textured surfaces that provided a convenient way to generate consistent, complex echo spectral patterns. Sandpaper coarseness is indicated by grit number, which is inversely related to the mean particle diameter of the abrasive coating. A two-alternative forced choice assay confirmed that free-tailed bats can discriminate between different sandpaper grits by echolocation (Smotherman et al., 2020), and based on this, we selected two sandpaper grits with distinctive echo spectral features that the bats readily distinguished. To make the acoustic stimuli, we ensonified three different reflecting surfaces [a flat Plexiglas surface, a 60-grit surface (265-μm mean particle diameter) and a 150-grit surface (92-μm mean particle diameter)] with an artificial downward FM sweep played through an ultrasonic loudspeaker while recording echoes with a microphone directly above the speaker. The sandpaper was always located at a distance of 80 cm, which produced a fixed temporal separation of 5 ms between the pulse and the recorded echo. This ensured that there was no temporal overlapping between pulse and echo and that the spectral notches caused in the echo were caused only by the surface structure of the sandpaper. Figure 1 shows the oscillogram, spectrogram, and the power spectra of the resulting echo mimic stimuli and their distinctive spectral notch patterns. For example, while the echo resulting from the flat surface shows no notches either in the spectrogram or the power spectra, the echo resulting from the 60-grit surface shows a 40-dB amplitude notch at 30 kHz and the echo from the 150-grit surface had two noticeable amplitude notches at 35 and 45 kHz, as well as a decreased amplitude at frequencies higher than 50 kHz.

### Increasing pulse repetition rate suppresses firing rate but enhances spike precision

**Figure 2.**
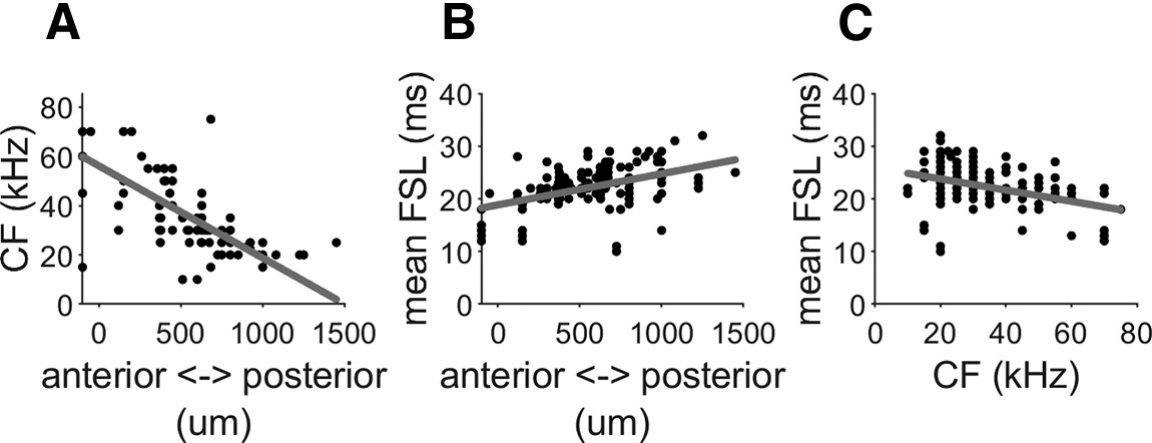
Topographical organization of the CF and the mean FSL in the A1 of the Mexican free-tailed bat. ***A***, CF as a function of the anterior-posterior location. ***B***, Mean FSL as a function of the cortical location. ***C***, Relation between CF and mean FSL. Details about the topographical and functional organization of the A1 in the Mexican free-tailed bat can be found in Macias et al. (2019).

Each stimulus pattern was presented as part of a pulse-echo combination delivered at three different repetition rates: 10, 12, and 15 per second. A 10-Hz sequence consisted of 10 pulse-echo combinations with a temporal separation of 100 ms. The 12-Hz sequence included twelve pulse-echo combinations with 88 ms intervals and the 15 Hz had fifteen pairs separated by 66 ms. Theses repetition rates were chosen based on our previous behavioral studies of pulse emission rates of this species of bat in a stationary position and while flying. Both stationary and flying free-tailed bats normally emit sustained emission rates of 10–15 pulses per second when actively echolocating (Smotherman et al., 2020). In total, we analyzed the activity of 165 neurons recorded from throughout the tonotopic axis of A1 in four bats. In addition to the sequences, each neuron was probed with changing sound pressure level and frequency to calculate the frequency response area. In each neuron, we measured the CF and the mean FSL at the CF 10 dB above the minimum threshold. The topographical organization of the CF (tonotopy) and the mean FSL of the A1 is provided in Figure 2. As described before (Macias et al., 2020), the A1 of the free-tailed bat is organized tonotopically with higher frequencies represented at rostral positions and a descendent gradient in the rostro-caudal direction (Fig. 2*A*). The neuronal mean FSL shows an inverse relationship with cortical locations, where shorter latencies are represented more rostrally and longer latencies are represented more in more caudal positions (Fig. 2*B*). Thus, there is an inverse relationship between CF and mean FSL in the A1 of the free-tailed bat (Fig. 2*C*).

**Figure 3.**
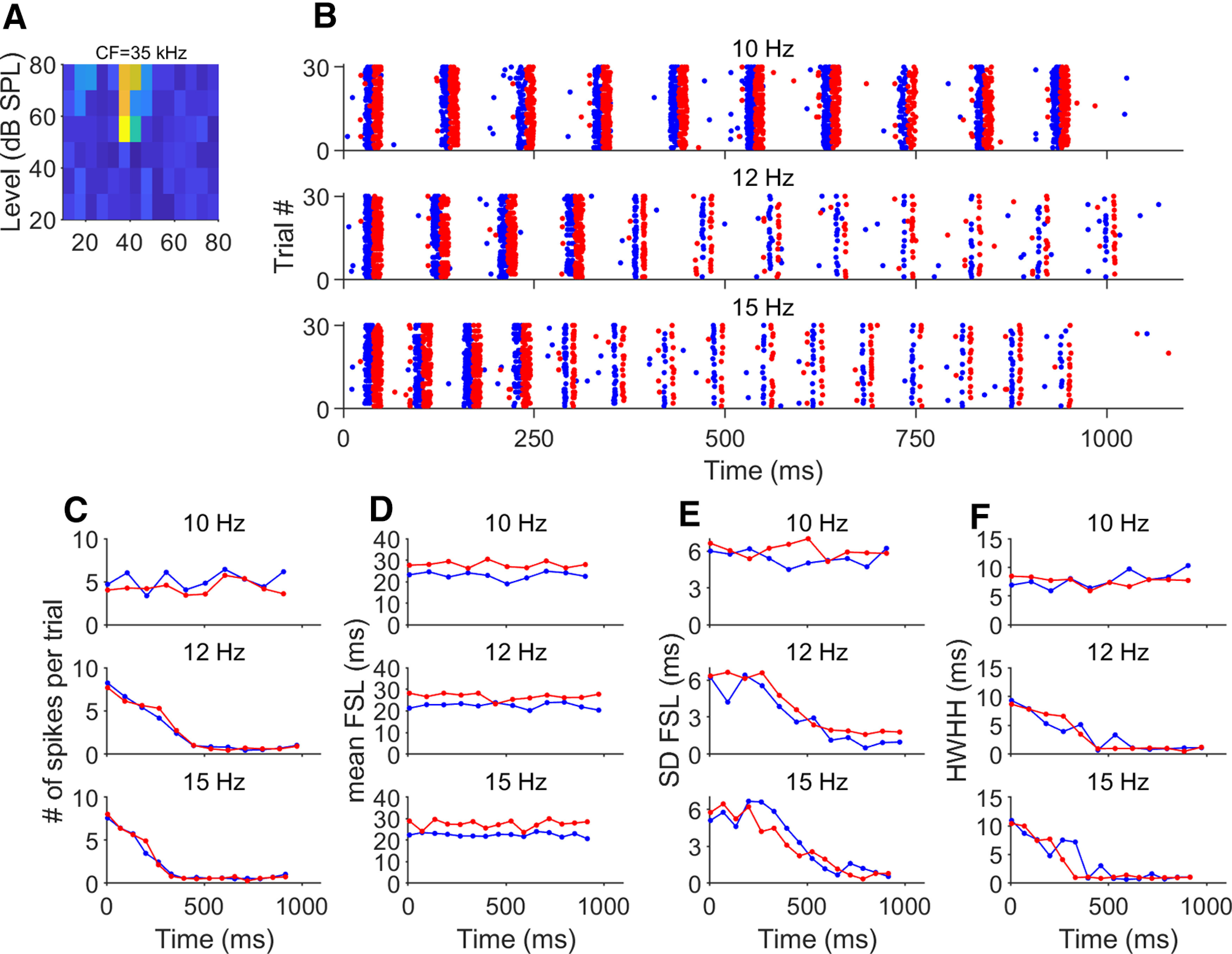
Example response to repetitive stimuli. ***A***, Frequency response area of an example neuron tuned with a CF of 35 kHz. The CF coincides with the frequency of one of the amplitude notches in the echo recorded from the 150-grit sandpaper. ***B***, Dot-raster display of the response of the neuron shown in ***A*** to three sequences of different repetition rate. In each response, blue dots represent the response to the flat surface and red dots represent the response to the 150-grit sandpaper. ***C***, Number of spikes as a function of time for the responses shown in ***B***. ***D***, Mean FSL as a function of time for the responses shown in ***B***. ***E***, Latency stability across trials calculated as the SD of the mean FSL as a function of time for the responses shown in ***B***. ***F***, Response precision calculated as the HWHH of the autocorrelation function of the PSTH as a function of time.

The time course of the response, as characterized by the number of spikes per stimulus during each sequence, was modulated by the pulse-echo repetition rate of the sequences. Figure 3 shows a neuron tuned to 35-kHz CF (see frequency response are in Fig. 3*A*) responding to the three different presentation rates of pulse-echo pairs reflected on the flat surface (blue dots) and the 150 grit (red dots). The response of this exemplary neuron to the 10-Hz sequence, as in all 165 recorded neurons, did not show a time-dependent change in the number of spikes evoked by each individual pulse-echo combination throughout the sequence. However, there was a progressive decrease in the number of spikes per stimulus across time in the responses to the 12- and 15-Hz sequences (Fig. 3*B*). The total number of spikes evoked by each sequential pulse-echo stimulus for this neuron in response to the three sequences for each surface are represented in Figure 3*C*. There was no change in the number of spikes in response to the 10-Hz sequence. However, repetition rates of both 12 and 15 Hz produced a significant decrease in the responses per pulse-echo across time, reaching a maximum effect within the first five pulses.

Surprisingy, the repetitive presentation of pulse-echo pairs at 12 and 15 Hz did not change the mean FSL. We calculated the mean FSL in response to each pair in the sequences and plotted these as a function of time (Fig. 3*D*). Note that this neuron’s response to the echoes from the 60-grit sandpaper (Fig. 3*B*, red dots, *D*, red line) had a longer response latency relative to the response to the flat spectrum (blue line). The longer latency at this frequency is a consequence of the amplitude notch at 35 kHz coinciding with the cells CF, thereby encoding the lower sound pressure level at this frequency (Macias et al., 2020).

Stimulus repetition rates of 12 and 15 Hz also had an effect on the latency stability (Fig. 3*E*). We calculated the SD of the FSL for each pulse-echo pair over the 30 trials (“SD FSL”) to estimate the temporal stability of the FSL response onset. When stimulating with the 10-Hz sequence, we saw no changes in the latency stability across time. However, in response to 12- and 15-Hz repetition rates we observed a decrease in the SD FSL, indicating a progressive increase in response first spike-time consistency during the stimulus sequence. As a measure of the temporal precision of the response to each pulse-echo, we calculated the half-width half-height (HWHH) of the autocorrelogram of the PSTH. The time course of the HWHH for this neuron is plotted in Figure 3*F*. In response to the 10-Hz sequences, there are no changes in the HWHH across time. However, when repetition rate is increased to 12 and 15 kHz, there is a progressive decrease in the HWHH.

**Figure 4.**
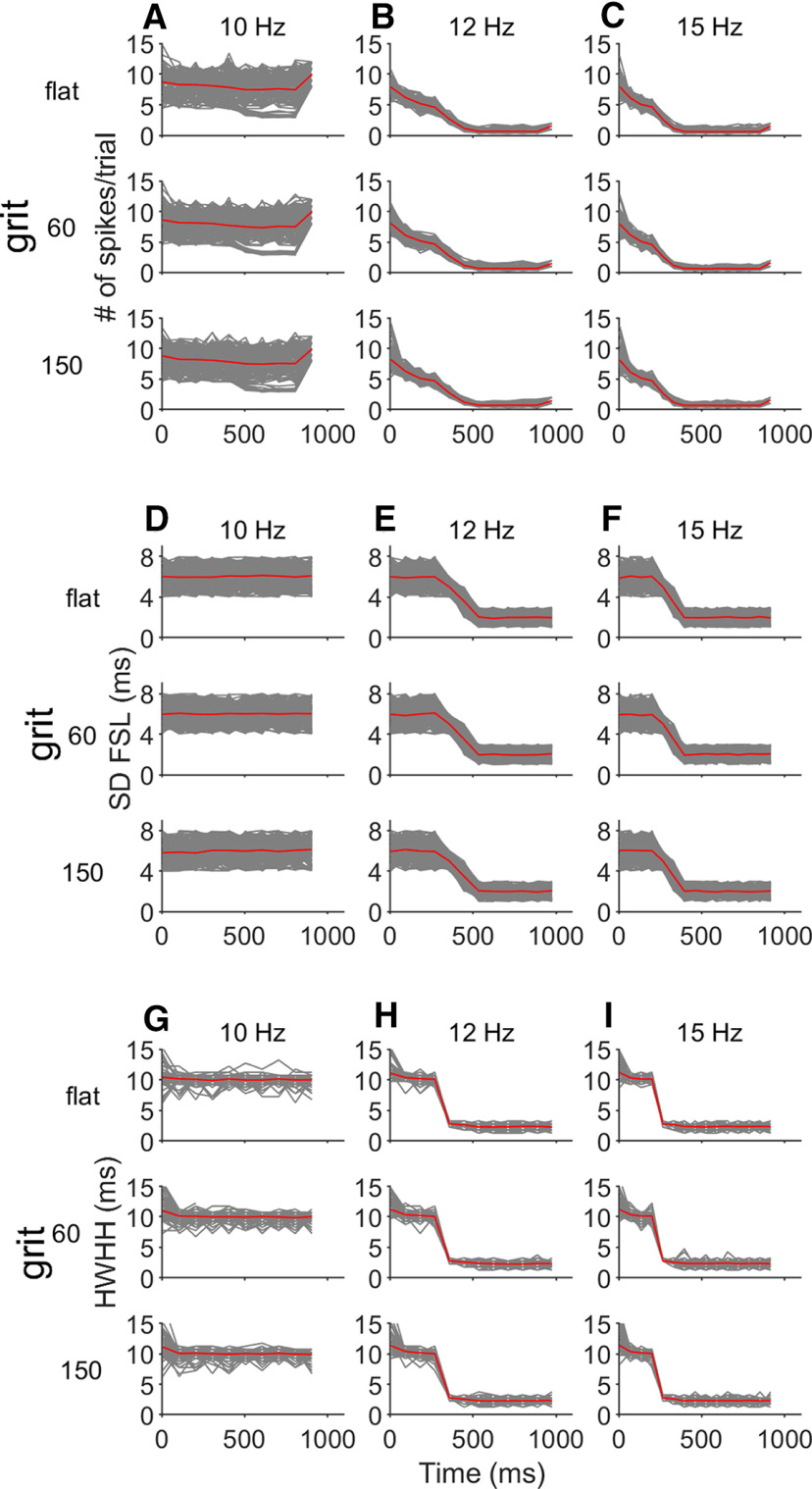
Responses to repetitive stimuli in the cortical neuronal population. ***A***, In response to the 10-Hz sequence, the number of spikes per trial did not change across time for any of the three surfaces (flat, 60- and 150-grit). ***B***, ***C***, There is a reduction of the number of spikes across time in response to the 12- and 15-Hz sequences for the three surfaces. Red line indicates the mean normalized number of spikes for all 165 neurons. ***D***, In response to the 10-Hz sequence, there are no changes in the latency stability across time for any of the three surfaces (flat, 60- and 150-grit). ***E***, ***F***, There is an increase of the latency stability (reduction of the SD of the FSL) across time in response to the 12- and 15-Hz sequences for the three surfaces. ***G***, There were no changes in the response precision (calculated as the HWHH on the autocorrelation function of the PSTH) across time in response to the 10-Hz sequence. ***H***, ***I***, Decrease of the HWHH in response to the 12- and 15-Hz sequences.

The changes in the number of spikes across time in the example neurons shown in Figure 3 were observed in the remaining 164 recorded cortical neurons (see Fig. 4*A–C* for the neuronal population). To evaluate whether the trend to decrease the number of spikes across time was statistically significant, we used the Mann–Kendall test (MKt). In this, an *H* value = 0 indicates no significant trend and a value of 1 indicates a significant increase or decrease. Overall, there was no change in the number of spikes in response to the 10-Hz sequence (MKt, *H* = 0, *p* = 0.37). However, repetition rates of both 12 and 15 Hz produced a significant decrease in the responses per pulse-echo across time (MKt, *H* = 1, *p* = 0.013 and *p* = 0.0022, respectively).

The effect of the stimulus repetition rates of 12 and 15 Hz on the latency stability on the neuronal population is shown in Figure 4*D–F*. When stimulating with the 10-Hz sequence, we saw no changes in the latency variability across time (MKt, *H* = 0, *p* = 0.474). However, in response to 12- and 15-Hz repetition rates we observed a decrease in the SD FSL (MKt, *H* = 1, *p* = 0.0004 and *p* = 0.0002, respectively). We observed a similar result for the temporal precision evaluated by the HWHH of the PSTH autocorrelogram (Fig. 4*G–I*). We found no variation of the response precision across time for the response to the 10-Hz sequences (MKt, *H* = 0, *p* = 0.27) and an increase in the responses to the 12 and 15 Hz (MKt, *H* = 1, *p* = 0.0026 and *p* = 0.0011, respectively).

### Fewer spikes improve SNR in neuronal synchronization profiles

As described previously (Macias et al., 2020), a longer response latency is produced when an amplitude notch is present in the echo spectrum at the neuron’s CF. This results in the neuron spiking in synchrony with neurons tuned to lower CFs and can thereby create a signature synchronization pattern across A1 representing the spectral envelope of a discreet echo and indirectly the physical properties (i.e., texture) of the reflecting object (Macias et al., 2020). We analyzed whether the decrease in number of spikes per echo together with the increase in response precision influenced the emergent spike synchronization patterns across A1. To do this, we reconstructed the topographical population dynamics from the CF-specific individual responses to each of the pulse-echo combinations and then calculated the corresponding spike synchronization matrices for all neurons. This allowed an evaluation of the changes in synchronization across time during the response to the different stimulus repetition rates. We evaluated spike train synchrony by using the spike-synchronization index (*c*), which quantifies the degree of synchrony from the relative number of quasi-simultaneous appearances of spikes (Kreuz et al., 2015; Satuvuori et al., 2017; Macias et al., 2020). Each matrix was calculated for each trial and from that, we calculated the evoked mean synchronization matrix for the responses to each pulse-echo pair.

**Figure 5.**
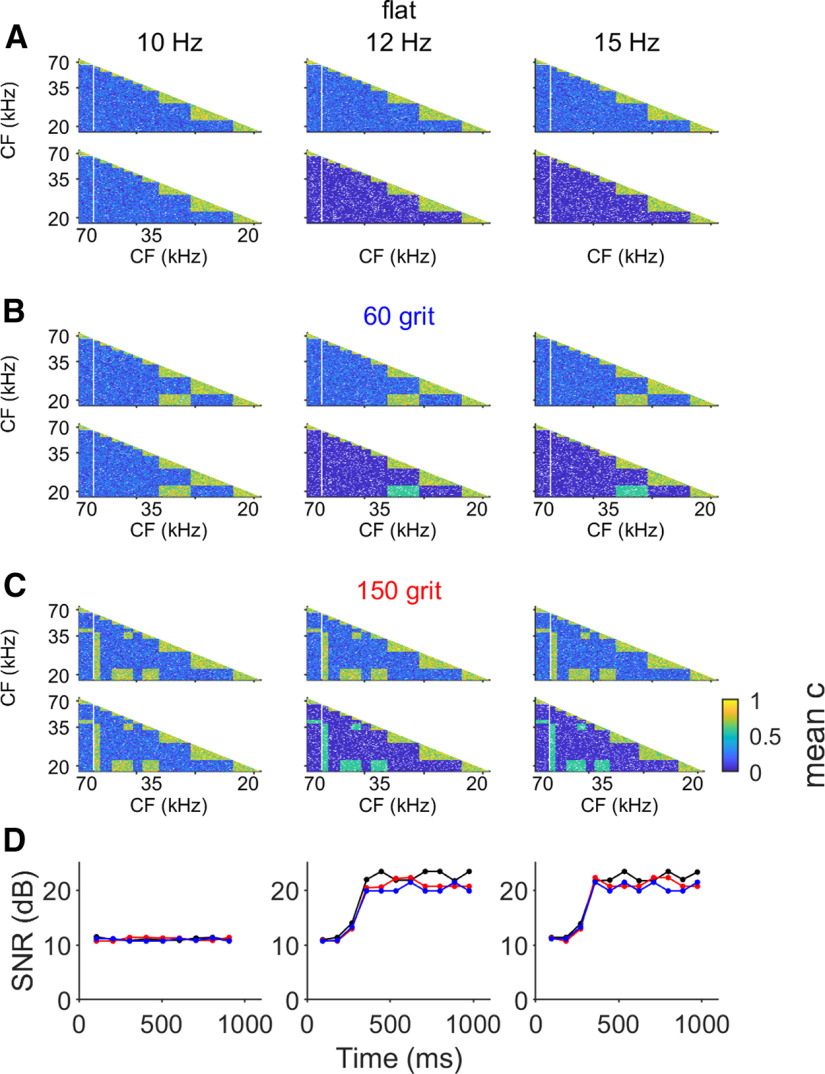
Sequences of pulses-echoes at high repetition rate increase SNRs of neuronal synchronization patterns. ***A***, Synchronization patterns calculated for the responses to the first and the tenth pulse-echo pairs from the flat surface of each repetition rate. ***B***, Synchronization patterns calculated for the responses to the first and the tenth pulse-echo pairs from the 60-grit surface of each repetition rate. ***C***, Synchronization patterns calculated for the responses to the first and the tenth pulse-echo pairs from the 150-grit surface of each repetition rate. All synchronization matrices were calculated for 155 neurons from four bats. ***D***, SNR calculated for each synchronization pattern in response to the three sequences. In each plot, black lines represent the response to the flat surface, red line represents the response to the 60-grit surface, and blue line represents the response to the 150-grit surface. SNR was calculated using the response to the first pulse-echo pair in each sequence as a reference.

The synchronization matrices calculated for all 165 neurons responding to the first and the tenth pulse-echo pair of the flat surface at all three presentation rates are represented in Figure 5*A*. In the synchronization matrices calculated for the response to the first stimulus at each repetition rates, there was greater synchrony (0.5–0.8) between neurons with the same CF and, as expected, fewer instances of synchronous firing (0–0.5) between neurons with different CF. During the presentation at 10 Hz, the synchrony values did not change throughout the pulse-echo sequences. However, at the 12- and 15-Hz repetition rates the synchronization rate between neurons with different CFs decreased over time, from <0.5 to <0.2 owing to fewer overall spikes and spurious coincidences, while the synchrony between neurons with same CF remained unaffected. Thus, the flat-spectrum echo became more clearly represented in the temporally coded population dynamics as stimulation rate increased.

In the synchronization matrices calculated for the responses to the 60- and 150-grit echo mimics there was an increase in synchrony between neurons tuned to the interference notches and those tuned to lower CFs (Fig. 5*A*,*B*), creating distinctive and prominent synchronization maps for each sandpaper echo. Background synchrony values continued to diminish throughout the sequence, leading to a steady increase in the SNR of the matrices. We quantified this by considering each matrix as an image and computed the corresponding SNR, in decibels, using the matrix calculated for the response to the first pulse-echo pair as a reference (Sage and Unser, 2003; Gonzalez and Woods, 2008). The time course of changes in SNR for each surface and each repetition rate are shown in Figure 5*D*. For all surfaces, there were no changes in SNR in the sequences with 10-Hz repetition rate, but there was an increase in the SNR across time at 12 and 15 Hz. This indicates that the decrease in mean spike numbers accompanied by an increase in spike-timing precision produced higher resolution synchronization maps, supporting better perceptual discrimination of echo spectral envelopes.

### Higher call rates provide more information about spectral details

**Figure 6.**
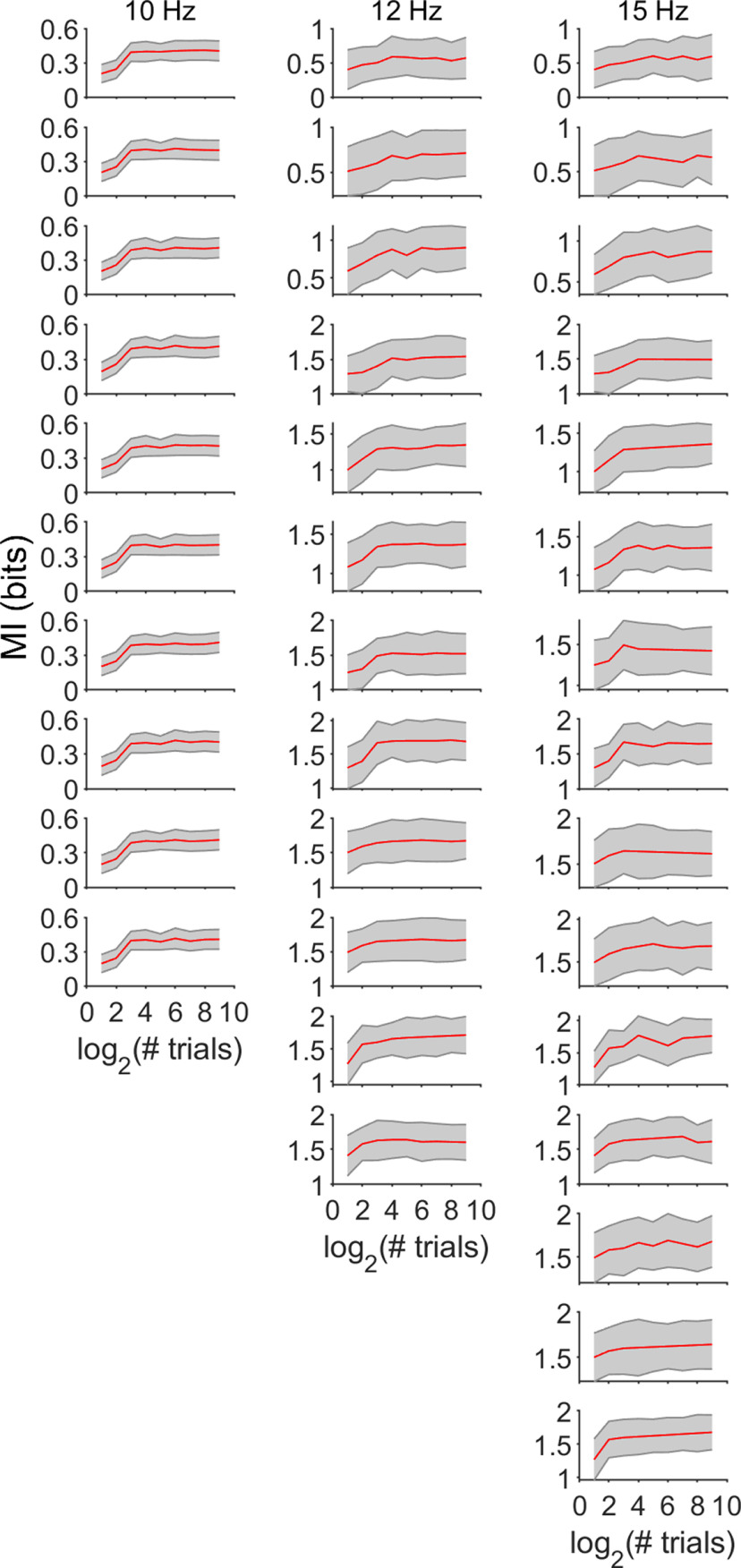
MI and the effect of sample size (# trials). Performance for bias-correction method used to calculate information values. Figure shows the data from the responses of neurons tuned to 30 kHz. Data were generated with statistics derived from the real experimental data to assess whether the number of trials included was sufficient for accurate calculation of MI). Calculation of MI was accurate at >15 trials, which was less than the number (30) used in our study.

**Figure 7.**
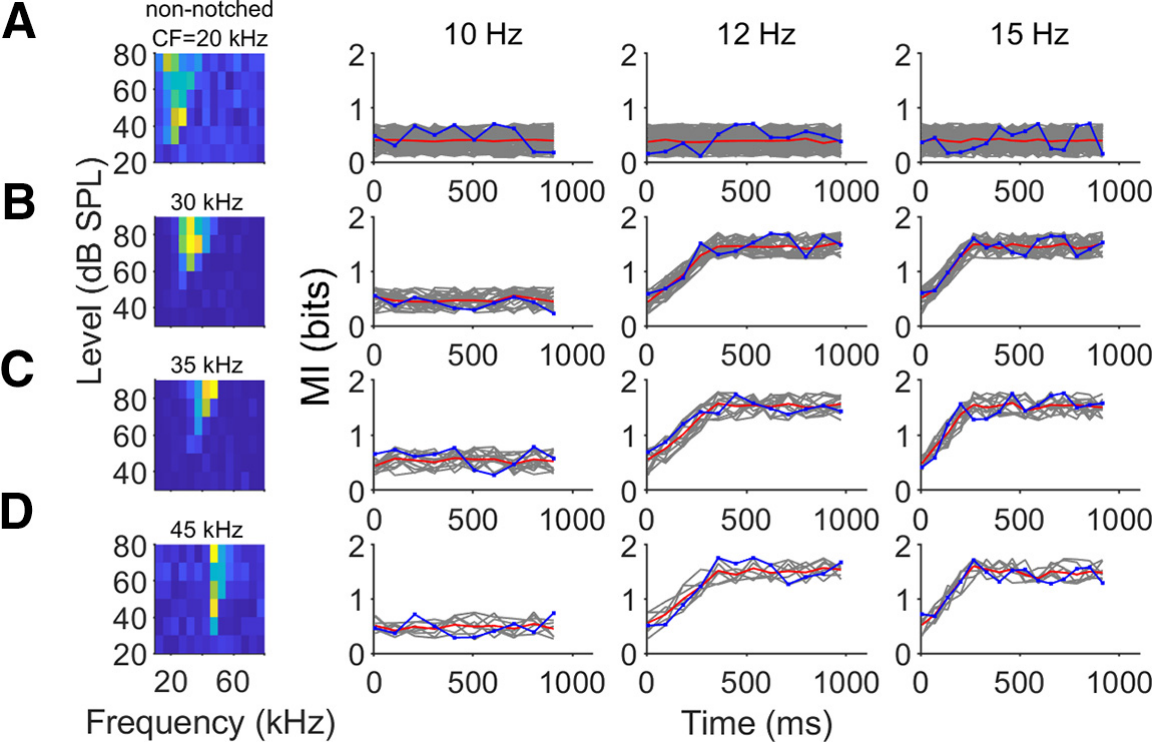
MI increases across time. ***A***, Non-notched neuron (CF does not coincide with the frequency of any notch). ***B***, Example neuron with CF = 30 kHz. ***C***, Example neuron with CF = 35 kHz. ***D***, Example neuron with CF = 45 kHz. MI across time for each example neuron is represented in blue. Gray lines represent MI across time for all the recorded neurons. Red line indicates mean MI for all neurons in each group.

To assess how changes in response precision influenced the amount of information carried by the mean FSL about the surface structure, we computed the MI between each individual pulse-echo pair and their respective neural responses. All analyses were based on a poststimulus window of 50 ms and each response window was subdivided into 2-ms bins. We evaluated the performance of the bias-correction methods used to calculate information values, by generating data with statistics close to the real experimental data and estimated the information in the neural codes following procedures used in previous studies (Panzeri et al., 2001; Macias et al., 2020). For each poststimulus window, information was underestimated when fewer than 16 trials were used (Fig. 6). However, considering the number of trials used in our recordings (30), the bias is small and does not affect the MI calculation. To calculate MI for individual neurons, we grouped the neurons according to their CF and its relationship to the frequency of the amplitude notches derived from the sandpaper interference patterns. Neurons were clustered in four groups (non-notched, 30, 35, and 45 kHz). Non-notched refers to those neurons where the CF did not overlap in frequency with any echo amplitude notch. The frequency response areas of four example neurons are depicted in Figure 7. In this example neuron as well as in the remaining units, there were no changes in MI across time in response to any of the sequences for the non-notched group (Fig. 7*A*). In the remaining sets (Fig. 7*B–D*), there was no change in the MI in response to the 10-Hz sequence, however, repetition rates of 12 and 15 Hz produce an increase in MI across time. Our MI calculations demonstrate that increasing pulse repetition rate would help an external observer more reliably classify and identify the reflecting object surface that created the echo. The reasons for this were 2-fold: reduced noise and increased latency stability and spike time precision. The reduced firing rate that occurred at higher stimulus repetition rates because of forward suppression paradoxically leads to more information about fine acoustic features embedded in the echo, which in this case relates to the target object’s shape.

## Discussion

This study characterized how the temporal pattern of acoustic stimuli affected biosonar information coding in the bat A1. Bats and cetaceans emit very brief broadband signals at high repetition rates to perform echolocation, one of the fastest and most precise examples of sensorimotor integration known for vertebrates (Grinnell, 1989). During flight, bats actively modulate their pulse emission rates, and here we show that in the auditory cortex, neuronal response dynamics were very sensitive to pulse repetition rate, with the main effect being fewer but more temporally precise action potentials occurring at higher repetition rates. Surprisingly, faster repetition rates had no impact on cortical neuron first-spike latency, instead producing an increase in the spike temporal precision and consequently an increase in MI about stimulus identity. Reduced overall firing at the population level lowered the SNR across the neuronal ensemble synchronization patterns and, hence, comprehensively supported better auditory object discrimination.

The forward suppression of auditory neurons that occurs in response to repeated stimulation is thought to result from a combination of synaptic depression and engagement of inhibitory circuits in A1 (Wehr and Zador, 2005; Bayazitov et al., 2013). This is generally thought to create a sensitivity for novel stimuli and context by suppressing responses to sustained background stimulation. Some of the proposed functional roles for forward suppression are that it contributes to cortical gain control (Ohzawa et al., 1982), enhances stimulus discriminability (Müller et al., 1999), maximizes information transmission by matching the coding strategy to stimulus statistics (Fairhall et al., 2001) and emphasizes new and interesting sounds (Ulanovsky et al., 2003; Malmierca et al., 2009). Reduction or total suppression of the cortical neuronal response at high repetition rates has been described not only in bats but also in rodents, birds and monkeys (Amin et al., 2004; Bartlett and Wang, 2005; Wehr and Zador, 2005; Narayan et al., 2007; Bayazitov et al., 2013; Schneider and Woolley, 2013; Zhou and Wang, 2014; Beetz et al., 2016; Hechavarría et al., 2016). In rats, cats and monkeys the neuronal response in the auditory cortex may be completely suppressed (Ulanovsky et al., 2004; Wehr and Zador, 2005; Bayazitov et al., 2013). Similarly, in response to natural acoustic sequences, neurons in the auditory cortex of the fruit-eating bat *Carollia perspicillata* showed an initial response to the first acoustic elements before they were strongly suppressed (Beetz et al., 2016; Hechavarría et al., 2016). In *Carollia*, it was thought that this suppression allowed for a more precise extraction of target distance information (Beetz et al., 2016), acting as a physiological filter that operates in the time domain to ensure sharp target-distance tuning and a more distinct topographic organization of echo delays. Furthermore, the receptive fields of target-range (pulse-echo delay-tuned) neurons in fruit bat auditory cortex became sharper with increasing repetition rate (O’Neill and Suga, 1982; Wong et al., 1992; Tanaka and Wong, 1993). Recent computational models of animal biosonar proposed that the bat auditory cortex is likely to encode echo spectral details by transposing amplitude-latency trade-offs in the ascending auditory system into a topographical profile of spike time registrations (Simmons, 2012; Ming et al., 2021). Here, we show that in insectivorous bats, the forward suppression induced by increasing pulse repetition rate sharpened the resolution of these time registrations, and thereby enhanced the cortical ensemble representation of echo spectral envelope, which may be particularly important for bats hunting insects on the wing.

During an attack on a flying insect or while approaching an obstacle, bats increase their pulse emission rates (Griffin, 1958; Schnitzler and Kalko, 2001; Ratcliffe, 2009). Increasing call emission rates generates more frequent information updates about the structure and position of the target (Moss and Surlykke, 2001; Ratcliffe, 2009; Moss and Surlykke, 2010; Elemans et al., 2011). In these experiments we found that as pulse emission rate increased, the auditory cortical substrate captured more information from each echo in the form of spike times about the reflecting surface structure. This suggests that when echolocating bats increase pulse repetition rate, they not only increase the rate of information flow but also the quality of that information by sharpening the cortical representation of the echo spectrum (Ulanovsky et al., 2003; Malmierca et al., 2009). The A1in the bat leverages the effects of forward suppression to improve their discrimination of auditory objects representing physical objects of different shape and structure.

Our experiments were conducted under pentobarbital anesthesia. Pentobarbital is known to decrease spontaneous activity in the cortex through a facilitation of IPSPs which may accelerate or enhance forward suppression dynamics. However, the mean time course of forward suppression observed in these experiments was similar to that described in awake primates (Fishman et al., 2001; Micheyl et al., 2005; Zhou and Wang, 2014; Malone et al., 2015). Recordings of neural discharges from awake guinea pigs (Creutzfeldt et al., 1980) and recordings of intracortical slow-wave activity in cats (Etholm et al., 1976) have documented that a significant amount of forward inhibition contributes to response dynamics in the awake preparation. Furthermore, Hechavarría et al. (2016) reported that they found similar suppression dynamics in the AC of both anesthetized and awake bats. Nonetheless, our interpretation of the temporal response characteristics of cortical cells obtained in this study must be presumed to include contributions from anesthesia. In addition, our observations in anesthetized animals exclude the possibility of the role played by attention when animals are actively producing sounds. Additional experiments will be required to disentangle the contributions of pentobarbital from the stimulus-driven inhibitory effects described here. Although recordings in an actively vocalizing bat would be most ideal to fully understand the neuronal response properties of the auditory cortex, the present study still offers valuable new insights on how temporal arraignment of sounds affects the cortical circuits processing behaviorally relevant stimuli.

## Synthesis

Reviewing Editor: Christine Portfors, Washington State University Decisions are customarily a result of the Reviewing Editor and the peer reviewers coming together and discussing their recommendations until a consensus is reached. When revisions are invited, a fact-based synthesis statement explaining their decision and outlining what is needed to prepare a revision will be listed below. The following reviewer(s) agreed to reveal their identity: Julio Hechavarria.

Both reviewers agree that the physiology in this study is very good. Three main points for the authors to consider are 1) the title does not represent what was done in the study as the bats were not actively calling, 2) potential bias in the MI calculations should be addressed by describing any form of bias-correction in the MI calculation and the number of trials, and 3) a discussion of anesthesia is necessary as there is a growing literature indicating that anesthesia potentiates forward suppression.

Reviewer 1:

The study describes a series of experiments conducted in anesthetized, passively listening bats, using semi-ecologically relevant sounds. The main result is that presenting fast sound sequences drives a forward suppression that renders temporally sharp neuronal firing in the bat AC. Such sharpening increases the amount of information that can be readout of cortical responses. Suppression also desynchronizes responses of neurons with different receptive fields.

These are interesting results that are framed within a neuroethologically clear question, namely, how does the bat brain cope with spectral notches present in biosonar echoes. While interesting, I think the article could profit from major changes in the introduction and discussion sessions. For example, the hypothesis is not easy to follow. Also, the discussion completely fails to address the issue of anesthesia and passive listening. In addition, the results part dealing with information theory needs clarification in several aspects. Below are my specific comments.

Comments:

Title: I don’t think the title does justice to what is being shown in the article. Neural signals were not recorded in vocalizing animals, so we really don’t know if similar results are going to be obtained during behavior. I know recording from vocalizing bats is not an easy task, so I am not asking for it, but I do encourage the authors to rethink their title.

Abstract: please indicate whether the animals were awake or anesthetized. Anesthesia can enhance forward suppression as shown in many studies in species other bats (Zador lab, Schreiner lab etc...).

Significance statement:

"auditory cortex neurons are unable to follow train of sounds” as it treads this statement is just not true. AC neurons can track fast sound streams even though most of them (especially during anesthesia) do stop tracking at ∼10-20 Hz repetition rate. Please rephrase.

Intro:

"Auditory cortex neurons respond only transiently to sounds. This forward suppression of neural activity” I don’t think responding transiently is a consequence of forward suppression. Please rephrase

"The bat auditory cortex appears biased towards time coding strategies due to the rapid and temporally precise nature of echolocation (Suga, 1989)” Again, I think this a controversial statement. The whole literature on delay tuning is basically pointing towards rate coding. Or perhaps it should be better clarified what is meant here by temporal and rate coding?

L50-66. The rationale described here is interesting but as written it is a tad difficult to follow. Conceptually speaking, the neural synchronization that putatively underlies notch discrimination occurs in the microsecond time scale. Forward suppression, in the other hand, occurs in the millisecond - seconds realm. More importantly, forward suppression does not need to affect the temporal tracking abilities of the neurons, i.e. a neuron can be suppressed and still fire. Can you please clarify what the hypothesis is and set the conceptual basis for it more explicitly? I think the question at hand is potentially very interesting but difficult to follow if concepts are not treated carefully. Also, I’m left wondering if there have been no studies in bats that already looked into forward suppression and temporal tracking in the auditory cortex. If there are they probably should be mentioned in the introduction because they can help to better build the hypothesis.

Methods

Lines 105-106: “Slightly anesthetized” Please define this, as anesthesia plays a major role on temporal processing.

"synchronization matrices” This is first mentioned in page 7. Can you clarify what these are?

P9- What was the distance between the target (i.e. the ensonified surface) and the speaker? Why did you choose 10, 12 and 15 Hz for the sequences?

L242: was the p= 0.013 for both rates?

Spike precision: I guess what is meant here is the latency stability or consistency. Precision typically includes the temporal sharpness of the response, at least as defined in studies by Panzeri and others. Calculating the half-width half-height of the PSTH’s autocorrelation function might help. Would be interesting to know if responses become temporally sharper as repetition rate increases.

MI and latency calculations. That latency SD and MI change over time when sequences are faster (12 and 15 Hz conditions) is indeed interesting. I am just wondering about the technical aspects of the calculations. It is not clear what MI is telling here. From what is written in the results, the responses were subdivided into 50 ms windows and within these windows, small epochs of 2 ms were created to calculate a sort of local MI corresponding to each 50-ms window. Is this correct? If so, why 2 ms? Would MI increase if shorter windows are considered? This might be particularly important when dealing with the temporally sharp notches. Second and most importantly, was there a form of bias-correction in the MI calculation. Was the number of trials used sufficient to reach a plateau in the information estimation curve? Third, the MI results can be nicely complemented with a precision estimation that does not rely on FSL. Precision can be better measured in a more accurate manner with the PSTH autocorrelation function. You can also provide an estimate of response duration and how it changes with repetition rate. MI is by far a better method, but it is just sometimes hard to grasp the meaning of it unless explained clearly.

Figures: How were the colors blue and red in the raster assigned? Why are there sometimes red dos occurring on top of the blue ones?

Discussion

"However, the functional organization of auditory system is different in frugivorous vs insectivorous bats” To my knowledge there is no compelling evidence about this. There is certainly a difference in behavior, but the echolocation machinery is C. perspicillata and P. discolor looks very similar to that of FM insect eating bats.

Anesthesia: It is disappointing that there is no mention of the relation between anesthesia and forward suppression. This study was conducted in anesthetized animals. One wonders whether suppression might be weaker if the animals were awake. Moreover, when animals are actively producing sounds, attention could play a role, and upstream areas might in fact control the amount of inhibition that drives forward suppression. These are likely frontal areas that could influence activity in AC whether directly or indirectly. I don’t think one has to discuss much about frontal cortices here, but that results might look different if the animals were awake and/or actively vocalizing should be mentioned.

Reviewer 2:

The premise of the research reported in this manuscript is that auditory “objects” are formed from neural spike responses that occur synchronously across subpopulations of cortical neurons that have different feature profiles, or are tuned to specific ranges along these feature dimensions. For detection of isolated sounds occurring at low repetition rates and not embedded in a surrounding auditory scene, each cell responds with multiple spikes, whose collective occurrence facilitates detection. For sounds that might be surrounded by other, interfering sounds, or when the repetition rate of the sounds is higher, the response pattern shifts from multiple spikes to single spikes, and the latency variability of these spikes shrinks to improve their ability to be detected by their synchronization to similarly reduced spikes in other neurons. The secret of the success in testing this hypothesis is that echolocating bats exhibit behavior consistent with the premise. The authors found that, indeed, spike rate coding (i.e., numbers of spikes) transitioned to a spike timing code when sound repetition rate increased from 10 Hz to even slightly higher rates of 12-15 Hz. The bats (free-tailed bats) emit 70-20 kHz downsweeping FM broadcasts and listen for echoes. For long range detection, pulse repetition rate is low, and target detection depends on numbers of spikes, most likely at the lowest frequencies of 2-30 kHz favored by lower atmospheric attenuation. Once a target (insect) is detected, the bat approaches to intercept it, repetition rate increases, and the task becomes one of localizing it in range and direction, and characterizing the target by its shape. These require accurate estimation of echo delay and the timing of different frequencies in the FM sweep of echoes. These, in turn, depend on registering response synchrony across cells tuned to different frequencies and local spectral shape. Replacing multiple spikes with single spikes and reducing their latency scatter is essential to achieve the more complicated processing that follows simple detection. The authors document their successful test of the hypothesis by computing information gain that contributes to completing the latency-based tasks. It is an excellent contribution, good physiology all around.
